# Pressure‐dependent adaptation strategies implied by the dissimilatory iron reducer *Orenia metallireducens* Z6

**DOI:** 10.1002/mlf2.70070

**Published:** 2026-02-19

**Authors:** Shuyi Li, Jiahao Pei, Jiasong Fang, Rulong Liu, Yuli Wei, Xianyu Huang, Guang Yang, Min Liu, Qin Lin, Robert R. Sanford, Hongbo Shao, Yongguang Jiang, Yidan Hu, Zhou Jiang, Qi Feng, Yu He, Chenxi Zhang, Yizhou Fan, Yiran Dong, Liang Shi

**Affiliations:** ^1^ School of Environmental Studies China University of Geosciences (Wuhan) Wuhan China; ^2^ Shanghai Engineering Research Center of Hadal Science and Technology Shanghai Ocean University Shanghai China; ^3^ State Key Laboratory of Geomicrobiology and Environmental Changes China University of Geosciences (Wuhan) Wuhan China; ^4^ School of Nuclear Science and Technology University of South China Hengyang China; ^5^ Shanghai Biozeron Biotechnology Co., Ltd. Shanghai China; ^6^ Department of Earth Science & Environmental Change University of Illinois Urbana‐Champaign Champaign Illinois USA; ^7^ Illinois State Geological Survey Champaign Illinois USA; ^8^ State Environmental Protection Key Laboratory of Source Apportionment and Control of Aquatic Pollution, Ministry of Ecology and Environment Wuhan China; ^9^ Hubei Key Laboratory of Yangtze Catchment Environmental Aquatic Science China University of Geosciences (Wuhan) Wuhan China; ^10^ MOE Key Laboratory of Groundwater Quality and Health, School of Environmental Studies China University of Geosciences (Wuhan) Wuhan China

## Abstract

Tolerance of high hydrostatic pressure (HHP) is the hallmark of deep subsurface microorganisms, while its mechanisms remain under‐investigated. This study explores HHP adaptation in the piezotolerant bacterium *Orenia metallireducens* across its near‐full pressure range (0.1–40 MPa). At inhibitory pressure (40 MPa), the organism redirected carbon flux toward more favorable energy generation and biosynthesis using ferric mineral as the “electron sink.” Furthermore, both universal and pressure‐dependent strategies enabled the organism to withstand varying pressures. These findings highlight the role of iron minerals in microbial HHP adaptation and reveal novel survival strategies, advancing our understanding of deep‐life evolution and biogeochemical impacts.

The deep subsurface, one of the Earth's largest microbial habitat, is characterized by high hydrostatic pressure (HHP) as an essential environmental stressor^1^, ^2^. HHP profoundly influences microbial life, affecting community structure, metabolic flexibility, and evolutionary adaptation. Research has revealed that HHP affects virtually all aspects of cellular physiology, including transcription, translation, membrane composition, multimeric protein assemblages, protein structure, and cellular motility^3^. While microbial adaptation to HHP is crucial for global biogeochemical cycles^4^, and microorganisms use strategies such as synthesizing osmolytes (compatible solutes) and activating antioxidant defenses^5^, ^6^, ^7^, research has historically focused on piezophiles, leaving common and ecologically important piezotolerant organisms underinvestigated^8^, ^9^. Among different microbial reactions, thermodynamic models indicated that microbial iron reduction was energetically more favorable under HHP compared to other electron‐accepting processes^10^. However, their specific adaptations to a pressure gradient and the role of ubiquitous iron minerals in this process remain poorly understood^5^. To address these gaps, we investigated the piezotolerant iron reducer *Orenia metallireducens* strain Z6, isolated from a 2.01 km‐deep terrestrial reservoir^11^, ^12^, across a hydrostatic pressure gradient from 0.1 to 40 MPa (Figure 1A). Our findings reveal a multi‐level, pressure‐dependent adaptation strategy that integrates physiological, metabolic, membrane, and genomic responses.

**Figure 1:**
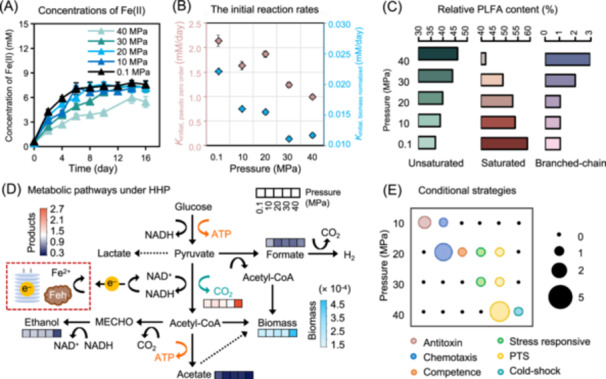
Multi‐level adaptation to a hydrostatic pressure gradient by *Orenia metallireducens* strain Z6. (A) Changes in Fe(II) concentrations over time under different pressures. As no significant increase in product concentrations was observed in the abiotic controls under the corresponding conditions, and thus, they were not illustrated. (B) Pseudo‐zero‐order Fe(III) reduction rates (pink diamonds, left axis) and biomass‐normalized Fe(III) reduction rates (blue diamonds, right axis) under different pressures. (C) Membrane adaptation. Relative abundance of phospholipid fatty acids (PLFAs) at different pressures. (D) Schematic diagram of metabolic pathways. In the heatmaps, the five cells (from left to right) correspond to the cultures at 0.1, 10, 20, 30, and 40 MPa, respectively. The dashed arrow indicates a feasible but potentially insignificant pathway. (E) Conditional transcriptomic strategies under different pressures. The size of bubbles indicates the number of genes that are pressure‐dependent and upregulated only under specific pressure(s). HHP, high hydrostatic pressure; PTS, phosphotransferase system.

Physiologically, strain Z6 showed stable iron‐reducing activity at 0.1–30 MPa but was significantly inhibited at 40 MPa (Figure 1A). In contrast, no biological iron reduction was observed in any of the abiotic controls across all pressure conditions, suggesting that iron reduction was mediated by enzymatic reactions (Figure S1). While the final Fe(II) production was reduced by approximately 25% at 40 MPa, the initial Fe(III) reduction rates showed a more pronounced decline, decreasing from 2.14 ± 0.12 mM/day at 0.1 MPa to 0.99 ± 0.04 mM/day at 40 MPa (Figure 1B). Protein analysis showed that biomass production peaked at 20 MPa (Figure S2), a pressure that closely aligned with its native habitat (~17 MPa)^12^. The optimal growth at its native pressure suggests a nuanced adaptation. While the rate of iron reduction per cell (biomass‐normalized rate) decreased at 20 MPa compared to 0.1 MPa, the organism compensated by accumulating significantly more biomass to maintain overall metabolic function (Figures 1B and S2).

A key physiological adaptation to HHP is the maintenance of cell membrane fluidity. Strain Z6 actively remodeled its membrane lipids in response to pressure (Figure 1C), a process known as homeoviscous and homeophasic adaptation^3^. Membrane analyses revealed phospholipid fatty acids (PLFAs) with 13–18 carbons in strain Z6, among which 15‐ and 17‐carbon species predominated (Table S1). As pressure increased, the proportions of unsaturated and branched‐chain fatty acids significantly increased, while the saturated fatty acid content decreased (Figure 1C and Table S2). Unsaturated and branched‐chain lipids have lower melting points and increase membrane fluidity, counteracting the pressure‐induced gelling or rigidity of the lipid bilayer, thereby maintaining essential cellular functions^13^, ^14^. Compared to their straight‐chain counterparts, branched‐chain PLFAs reduce melting temperatures and prevent the formation of crystalline structures between acyl chains, thereby further enhancing membrane fluidity under pressure^3^. This aligns with a previous study showing that the monounsaturated‐to‐polyunsaturated PLFA ratios for marine microorganisms correlate positively with their isolation depth^13^. Similarly, *Photobacterium profundum* SS9 requires monounsaturated fatty acids for growth at high pressure and low temperature^14^.

Critically, a metabolic shift under the inhibitory pressure (40 MPa) was observed (Figure 1D). Specifically, glucose consumption slowed, and the final concentrations of formate and acetate decreased significantly. While ethanol yield decreased, CO_2_ production and the final Fe(II)/glucose ratio increased significantly (Figures 1D and S3; Table S2). This indicates a pressure‐induced redirection of carbon and electron flux. Metabolic reconstruction revealed that strain Z6 decomposed glucose via glycolysis and pyruvate metabolism^11^. These pathways include two steps in glycolysis and one step in the downstream pyruvate metabolism (acetyl‐CoA to acetate) that generate ATP. Beyond energy production, pyruvate metabolism also regulates the NADH/NAD^+^ balance. Pyruvate‐to‐ethanol conversion consumes NADH to form NAD^+^, while pyruvate decarboxylation generates NADH along with the formation of C1 compounds (CO_2_ or formate) and acetyl‐CoA^15^. Our observed carbon flux redirection might be due to pressure‐induced inhibition of key enzymes such as alcohol dehydrogenase^16^. This hypothesis aligns with the earlier findings on *Clostridium paradoxum* ^16^. For this organism, elevated pressure attenuates ethanol production kinetics by reducing enzymatic activity, which is attributed to a pressure‐induced conformational state change characterized by lower substrate affinity for NAD⁺ and restricted hydride transfer^16^. Thus, a metabolic shift occurs, moving away from ethanol formation and toward pyruvate decarboxylation. This shift provides key advantages. It generates more acetyl‐CoA, which is a key biosynthetic precursor^15^. It also produces more reduced electron carriers (e.g., NADH), which then support enhanced ferrihydrite reduction by strain Z6. This mechanism highlights the central role of Fe(III) minerals as an “electron sink.” It reframes the conventional theory, which often regards ferric minerals in fermentation as merely incidental sinks. In contrast, our work suggests that they are active facilitators that modify electron and carbon fluxes, ultimately enhancing microbial growth under inhibitory HHP. While thermodynamic modeling predicts increased favorability of microbial iron reduction under HHP^10^, our calculations show that the changes in Gibbs free energy (Δ*G*) across our experimental pressure range are not significant (Table S2). Instead, the observed thermodynamic difference between the inhibitory pressure (40 MPa) and the relatively lower pressures primarily relates to the altered stoichiometry between glucose decomposition and iron reduction. By accepting excess electrons, the amended ferrihydrite actively facilitates this metabolic rerouting, allowing strain Z6 to optimize energy production and growth yield even under inhibitory pressure. This adaptation makes the overall metabolism thermodynamically more favorable at 40 MPa (Δ*G* = −361.12 kJ) than that under lower pressures (Δ*G* = −343.51 to −303.15 kJ) (Table S2), explaining how comparable biomass yields were maintained despite physiological stress.

Sequencing of the cDNA libraries for the pressure‐adapted cultures yielded 2.00–2.43 Gb of high‐quality reads per sample (Table S3). Compared to the gene expression under 0.1 MPa controls, 463, 1794, 2082, and 2059 genes were identified as differentially expressed genes (DEGs) (Figure S4). Transcriptomic analysis revealed the genetic basis for these integrated adaptations (Figures 1E and S5). We identified general pressure adaptation strategies based on the genes upregulated across all high‐pressure conditions (10–40 MPa) (Figure S5 and Table S4). These included universal stress response genes for: (i) antioxidant defense (e.g., the *grx* gene encoding glutaredoxin). Upregulation of antioxidant systems represents a universal bacterial defense strategy. HHP is known to induce oxidative stress; thus, activating these pathways is a critical strategy to maintain cellular integrity, similar to the upregulation of *gpx* and *katE* by *Shewanella piezotolerans* WP3 under HHP and cold stress^17^. (ii) DNA/protein repair. The gene encoding the LexA repressor, which represses SOS response genes, was upregulated. In contrast, *recA*, which is essential for homologous DNA repair, was downregulated. This may result from a negative feedback loop in the bacterial SOS response system, ensuring an appropriate repair response while preventing potential harm from an overactive one. For example, the *clpX* and *clpP* genes encoding ATP‐dependent Clp proteases critical for protein quality control were downregulated, which likely plays a key role in protein degradation, stress response, and cellular physiology regulation, perhaps as a mechanism to conserve cellular energy under stress. (iii) Ion transport. Upregulation of *afuB* and *fbpB* encoding putative ferric transporters highlights the critical challenge of iron homeostasis under pressure. The organism must acquire essential iron while managing the risk of oxidative stress from free iron. Thus, upregulation of these specific transporters suggests a fine‐tuned mechanism to balance metabolic need against potential toxicity. (iv) Compatible solute biosynthesis. Genes such as *gltB/D*, *proW/X*, and *proX*, encoding the proteins for glutamate, glycine, and betaine transport, respectively, were upregulated. These allow uptake or production of the organic piezolytes to stabilize proteins and membranes against pressure stress^6^. In addition, upregulation of the glutamate synthase genes (*gltB/D*) is particularly significant, as glutamate is a key piezolyte observed in many piezophiles (e.g., *Desulfovibrio* spp.)^7^.

More significantly, conditional pressure adaptation strategies on the specific pressure regime were identified (Figure 1E and Table S5). At 10–30 MPa, where active microbial growth occurred, strain Z6 upregulated the genes related to chemotaxis (e.g., histidine kinase) and natural competence^18^. The flagellar system is usually important for piezophilic growth, serving a critical function for nutrient acquisition and predator avoidance^3^. Nearly all the genes associated with flagellar synthesis and regulation in strain Z6, however, were downregulated at elevated pressures (Table S6). Thus, the upregulation of chemotaxis proteins under elevated pressures by strain Z6 suggests an alternative strategy for fine‐tuning environmental sensing. Interestingly, the genes related to the TonB system, which powers outer membrane transport at the cost of the proton motive force, were downregulated at 10–40 MPa compared to that at standard pressure (Table S4). This downregulation implies an active strategy to reallocate energy toward detecting favorable microniches for activating natural competence, potentially enabling nutrient recycling from lysed cells as an energetically efficient strategy. In contrast, at the inhibitory 40 MPa, a different suite of genes (e.g., those encoding the phosphotransferase system (PTS) and cold‐shock proteins) was overexpressed, indicating a shift to a “survival” posture (Figure 1E and Table S5). PTS is a central regulator for carbon metabolism and stress responses. Studies suggest that PTS may modulate oxidative stress tolerance or exert genetic regulation in non‐ or slow‐growing cells exposed to lethal stress^19^. In this study, it may function as a high‐affinity scavenging system or as a key sensor in a broader stress‐response cascade, helping the cells withstand inhibitory pressure while in a slow‐growing state. Meanwhile, the cold‐shock protein response confirms the known mechanistic linkage between adaptation to HHP and cold stress^19^.

The ecological relevance of these findings is underscored by the global distribution of the genus *Orenia*. A survey of isolated strains and metagenomic data showed their presence in diverse habitats ranging from surface hypersaline lakes to deep subsurface (Figure S6 and Table S7). These environments span the pressure range, aligning with that investigated in this study. This strongly suggests that the multi‐level, Fe‐dependent adaptive strategies observed in strain Z6 are not unique, but rather represent fundamental and ecologically significant survival mechanisms for this widespread genus across the shallow to deep biosphere.

In conclusion, *O. metallireducens* strain Z6 uses multi‐level and pressure‐dependent adaptation strategies under HHP conditions. The strategies are conditional: it maintains active metabolism (0.1–30 MPa) via chemotaxis and membrane remodeling, but shifts to a conservative “survival mode” at inhibitory pressure (40 MPa). This shift involves actively rerouting carbon flux away from ethanol and toward CO_2_/biomass to optimize energy generation. Crucially, we identify ubiquitous Fe(III) minerals not as passive acceptors, but as active “electron sinks” essential for this metabolic flexibility and enhanced biomass production under stress. Given the global distribution of the *Orenia* genus (Figure S6), this Fe‐dependent adaptation likely represents a fundamentally and ecologically significant survival strategy for the deep biosphere^20^.

## ETHICS STATEMENT

This article does not contain any study with human participants or animals.

## Supporting information

Supporting information.

Supporting information.

Supporting information.

Supporting information.

Supporting information.

## Data Availability

The transcriptomic data have been deposited in the National Center for Biotechnology Information (NCBI) GenBank under the BioProject PRJNA1211793.
