# Supplementary material for: Pressure‐dependent adaptation strategies implied by the dissimilatory iron reducer Orenia metallireducens Z6
Source: mLife. 2026 Feb 19;5(1):122–5. doi: 10.1002/mlf2.70070 (PMC12948478; doi:10.1002/mlf2.70070)
Supplement: Supplementary file 1 — Supporting information. [file MLF2-5-122-s001.docx]

Supplementary Information for

**Pressure-dependent Adaptation Strategies Implied by the Dissimilatory Iron Reducer *Orenia metallireducens* Z6**

Shuyi Li^1^, Jiahao Pei^2^, Jiasong Fang^2^, Rulong Liu^2^, Yuli Wei^2^, Xianyu Huang^3^, Guang Yang^3^, Min Liu^4^, Qin Lin^5^, Robert R. Sanford^6^, Hongbo Shao^7^, Yongguang Jiang^1^, Yidan Hu^1^, Zhou Jiang^1^, Qi Feng^1^, Yu He^1^, Chenxi Zhang^1^, Yizhou Fan^1^, Yiran Dong^1, 3, 8, 9, 10^*, Liang, Shi^1, 3, 8, 9, 10^

^1.^ School of Environmental Studies, China University of Geosciences (Wuhan), Wuhan 430074, China

^2.^ Shanghai Engineering Research Center of Hadal Science and Technology, Shanghai Ocean University, Shanghai, 201306, China

^3.^ State Key Laboratory of Geomicrobiology and Environmental Changes, China University of Geosciences, Wuhan 430074, China

^4.^ School of Nuclear Science and Technology, University of South China, Hengyang, 421001, China

^5.^ Shanghai Biozeron Biotechnology Co., Ltd, Shanghai, 201306, China

^6.^ Department of Earth Science & Environmental Change, University of Illinois Urbana-Champaign, Champaign, IL, 60801, U.S.A.

^7.^ Illinois State Geological Survey, Champaign, IL, 60801, U.S.A.

^8.^ State Environmental Protection Key Laboratory of Source Apportionment and Control of Aquatic Pollution, Ministry of Ecology and Environment, Wuhan, 430074, China

^9.^ Hubei Key Laboratory of Yangtze Catchment Environmental Aquatic Science, China University of Geosciences (Wuhan), Wuhan, 430074, China

^10.^ MOE Key Laboratory of Groundwater Quality and Health, School of Environmental Studies, China University of Geosciences, Wuhan 430078, China

*Corresponding author. Mailing address: 388 Lumo Road, Wuhan, China. Phone: +86-27-67883152. Email: [dongyr@cug.edu.cn](mailto:dongyr@cug.edu.cn)

The supplementary information consists of 33 pages, 8 tables, and 7 figures.

**Methods and Material**

Chemical Reagents and Mineral Preparation

Unless stated otherwise, the chemicals were purchased from Shanghai Macklin Biochemical Co., Ltd. or Sinopharm Chemical Reagent Co., Ltd. (Shanghai, China), with a purity of Analytical grade or higher. The ferric chloride (FeCl₃·6H_2_O) used for synthesizing ferrihydrite was purchased from Sigma-Aldrich (Shanghai) Co., Ltd. (Shanghai, China). Ultrapure water (18 MΩ) was used for preparing all the solutions. Ferrihydrite was synthesized following the method described by Cornell and Schwertmann^1, 2^.

Strain and culturing conditions

*Orenia metallireducens* strain Z6 was isolated in our previous studies^3, 4^. A series of batch experiments was performed to investigate the adaptation of strain Z6 under different hydrostatic pressures ranging from 0.1 to 40 MPa in 10 MPa increments. Strain Z6 was cultivated in the anaerobic tubes filled with modified synthetic groundwater medium (pH 7.0–7.2) and equilibrated with high-purity N_2_ (99.9%)^3^. The cultures were contained in custom-made anaerobic tubes (16 mL) sealed with butyl rubber stoppers and aluminum crimp seals. In addition, some cultures contained in 100 mL serum bottles were also prepared to analyze phospholipid fatty acid (PLFA) of the cells in response to varied pressure conditions.

Strain Z6 used for inoculation was pre-cultured in synthetic groundwater medium amended with 5 mM glucose and harvested after approximately 35 hours of incubation. The cells were inoculated into fresh medium with a dilution ratio of 1:20, leading to an initial OD_600_ of approximately 0.015 (~ 10^7^ cells/mL)^5^. Glucose was added from a sterile and anaerobic stock solution with a final concentration of 10 mM. Synthesized ferrihydrite (15 mM) was used as the electron acceptor. The remaining headspace in the anaerobic tubes was filled with the same medium inside an anaerobic glove chamber (Vigor Technologies (Suzhou) Co., Ltd, Jiangsu, China) filled with N_2_. In addition, a 2 mL syringe containing 1 mL medium was injected through the rubber stopper of the anaerobic tube before it was placed into the pressure vessels (Nantong Feiyu Petroleum Technology Development Co., Ltd, Jiangsu, China). The cultures were incubated at 37 ± 0.2℃ and under one of the selected hydrostatic pressure conditions (0.1, 10, 20, 30, and 40 MPa).

Sampling pretreatment and analyses

At each time point, a total of 2.2 mL of well-mixed culture was aseptically collected and processed as follows: (1) 1 mL was injected into a 10 mL sealed and N_2_-flushed serum vial containing 200 μL 50% phosphoric acid for dissolved inorganic carbon and H_2_ analyses; (2) 0.2 mL was promptly acidified with 0.2 mL 1N HCl to determine the concentrations of 0.5 N HCl-extractable ferrous iron (Fe(II)) using the ferrozine method^6^; (3) the remaining 1 mL culture was centrifuged at 8000×g and 4°C for 2 min, with the supernatant preserved in 500 μL 10 mM H_2_SO_4_ solution to measure the concentrations of glucose and soluble fermentation products using a high-performance liquid chromatograph (HPLC)^7^. The pellets were washed once with cold 1×PBS buffer solution (pH 7.4) and stored at -20°C to measure protein concentrations. An additional 100 mL cultures were processed after 48 hours for phospholipid fatty acid (PLFA) analysis using a gas chromatograph connected with a mass selective detector (GC-MS) (Agilent Technologies, Inc., CA, U. S.)^8, 9^. Based on reaction stoichiometries, thermodynamic analyses of Fe-facilitated fermentation by strain Z6 under varying HHP conditions were conducted using the method in our earlier study. In addition, 2 mL aliquots were collected for RNA extraction and transcriptomic analyses to evaluate gene expression profiles by strain Z6 at the different pressure conditions^10^.

Cellular and chemical analyses

The optical density at 600 nm wavelength (OD_600_) of the cultures was determined using a GENESYS 50 UV-Vis spectrophotometer (Thermo Fisher Scientific Inc., MA, U.S.). The 0.5 M HCl-extractable Fe(II) was analyzed with the ferrozine method at 562 nm^11^ using a microplate reader (Molecular Devices LLC, CA, U.S.). The initial rate constants for microbial iron reduction were calculated using a pseudo-zero-order model as suggested in our recent study^11^. The concentrations of glucose and soluble fermentation products were determined using the HPLC installed with a UV detector (Shimadzu Corporation, Kyoto, Japan) and an Aminex HPLC column (Bio-Rad Laboratories, Inc., CA, U.S.). The mobile phase was 5 mM H_2_SO_4,_ and the temperature was set to 55°C. The protein content was measured using a BCA Protein Assay Kit (Takara Biomedical Technology (Beijing) Co., Ltd., Beijing, China). The concentrations of CO_2_ and H_2_ were quantified with a gas chromatograph (GC) installed with a thermal conductivity detector (TCD) (Shimadzu Corporation, Kyoto, Japan).

Phospholipid fatty acid (PLFA) extraction and analyses

The cells for phospholipid fatty acids (PLFAs) analysis were collected from the cultures grown in the anaerobic bottles after 48 hrs of incubation under different hydrostatic pressures. The cultures were pooled and pelleted by centrifugation at 8000 g for 5 minutes. The pellets were freeze-dried overnight and extracted for phospholipids following the protocol suggested by Bligh and Dyer (1959)^8, 9^. The retrieved phospholipids were subject to derivatization through mild alkaline methanolysis to produce phospholipid fatty acid methyl esters (FAMEs)^12^. For quantification, an internal standard (n-C18 fatty acid) was added before transesterification. The FAMEs were analyzed using an HP 6890 Gas Chromatograph equipped with an HP 5973 Mass Selective Detector (GC-MS) and a DB-5 MS capillary column (30 m × 0.25 mm internal diameter, 0.25 μm film thickness) (Agilent Technologies, Inc., CA, U. S.). The program for the GC was: 50°C for 1 minute, then increased to 210°C at a rate of 2°C per minute, and finally increased to 310°C at a rate of 4°C per minute with a 15-minute hold. The compounds were identified by comparing the obtained mass spectra with the published references^13^ using the MIDI identification software (MIDI Inc., DE, U.S.).

RNA-Seq analyses.

To understand the adaptive strategies employed by strain Z6 in response to pressure changes, the samples were collected at the 48^th^ hour, when they were in the exponential phase, based on the growth experiments. The genomic DNA and RNA for strain Z6 under different hydrostatic pressures were extracted using the TRIzol^®^ Reagent (Thermo Fisher Scientific Inc., CA, U.S.) according to the manufacturer’s instructions. The genomic DNA was removed using DNase I (Takara Biomedical Technology Co., Ltd., Japan). Then the RNA quality was determined using a Qubit 2100 Bioanalyzer (Agilent Technologies, Inc., CA, U.S.). The strand-specific RNA-seq libraries were prepared using the TruSeq RNA Sample Preparation Kit (Illumina, San Diego, CA) with 5 μg of total RNA. Briefly, rRNA was removed using the Ribo-Zero rRNA Removal Kit (Illumina, San Diego, CA), which was followed by RNA fragmentation. The cDNA synthesis, end repair, A-base addition, and ligation of Illumina-indexed adapters were performed according to the manufacturer's protocol. The libraries were size-selected for the cDNA fragments of 200–300 bp on a 2% low-range ultra-agarose gel and then amplified by PCR using Phusion DNA Polymerase (New England Biolabs, MA, U. S.) for 15 cycles. After quantification with TBS380, the paired-end libraries were sequenced with an Illumina NovaSeq 6000 sequencing at Shanghai BIOZERON Co., Ltd. For each pressure condition, triplicate samples were extracted and sequenced.

The raw paired-end sequencing reads were quality trimmed with Trimmomatic (v.0.36) with the parameters SLIDINGWINDOW:4:15 and MINLEN:75^14^. The clean reads were then aligned to the reference genome of strain Z6 with the orientation mode of Rockhopper^3, 15^. To identify differentially expressed genes (DEGs) between the samples under elevated and standard hydrostatic pressures, the expression level for each gene was calculated as transcripts per million mapped reads (TPM). The fold change (FC) value for each gene was calculated by dividing its TPM under the gene under specific pressure conditions by that under 0.1 MPa. Differential expression analysis was performed using edgeR^16^. To understand the functions of the DEGs, the overexpressed genes were annotated based on Gene Ontology (GO) and KEGG databases using Goatools^17^ and KOBAS^18^, respectively. The DEGs were considered significantly enriched when their log_2_(FC)≥1 and Bonferroni-corrected *p*-values were ≤0.05.

To validate the RNA-Seq data, reverse transcriptase-PCR (RT-PCR) was performed on 3 genes of 12 randomly selected samples. The primers for the targeted genes were designed using NCBI Premier-BLAST (https://www.ncbi.nlm.nih.gov/tools/primer-blast/), and the details for the primer sets are listed in Table S8. The TB Green® Premix Ex Taq™ II FAST qPCR reagent kit (Takara Biomedical Technology (Beijing) Co., Ltd., Beijing, China) and a BioRad CFX96 Real-time PCR System (BioRad Laboratories, CA, U. S.) were used for the RT-PCR. The fold changes in RNA-Seq and RT-PCR were plotted, and the correlation coefficient (R²) between the two datasets was calculated.

Calculation of Gibbs free energy

The stoichiometric oxidation-reduction equations were constructed following the established methodology^5^. The standard and final Gibbs free energies for these chemical equations under various high hydrostatic pressure (HHP) conditions were calculated (Eq. S1). Given the absence of headspace in our system, we first needed to determine whether CO₂ under 40 MPa existed in a supercritical state and obeyed Henry’s law (Eq. S2). The supercritical point for CO₂ is achieved at 37°C only when the pressure exceeds 7.38 MPa. Our measurements showed a maximum CO_2_ concentration of 24 mM, corresponding to a partial pressure of CO_2_ (*P*_CO2_) of 72.4 kPa, which was well below the supercritical threshold. This confirmed that no supercritical CO_2_ phase formed in our reaction system. Accordingly, we applied experimental temperature corrections for our experimental conditions (37°C or 310.15 K) using the Gibbs-Helmholtz equation (Eq. S1).

| $\Delta G=\Delta G^{o}+RTln(\frac{P}{P^{o}})$ | Eq. S1 |
| --- | --- |

Where $\Delta G^{o}$refers to the Gibbs free energy under standard conditions (298 K, 0.1 MPa), while $\Delta G$ represents the Gibbs free energy under the experimental conditions (310.15 K). T_2_​ and T_1​_ indicate the experimental temperature and the standard temperature, respectively.

| $P_{{CO}_{2}}=\frac{C}{K_{H}}$ | Eq. S2 |
| --- | --- |

Here, *P_CO2_* represents the partial pressure of CO₂, *C* denotes the concentration, and $K_{H}$is the Henry's constant.

Under isothermal conditions, the volume change ($\Delta V$) during the reaction process can be considered negligible since the anaerobic tube system experienced minimal volume variations. Consequently, the pressure change (dP) approaches zero, making the $\Delta VdP$ term effectively zero in our calculations (Equations S3-S4). This negligible pressure-volume work means the effect on the Gibbs free energy change ($\Delta G$) by hydrostatic pressure can be reasonably omitted in our experimental system.

| $dG=VdP-SdT$ | Eq. S3 |
| --- | --- |
| $\Delta G=\Delta G^{0}+\int_{P^{0}}^{P} \Delta Vdp$ | Eq. S4 |

Consequently, the reaction free energy at various pressures was calculated by accounting for the entropy (*ΔS*) and enthalpy (*ΔH*) changes of the reaction components at 37 °C (310.15 K) as detailed in Equations S5-S7.

| $\Delta H\left( T_{2} \right)=\Delta H\left( T_{1} \right)+\int_{T_{1}}^{T_{2}} C_{P}dT$ | Eq. S5 |
| --- | --- |
| $\Delta S\left( T_{2} \right)=\Delta S\left( T_{1} \right)+\int_{T_{1}}^{T_{2}} \frac{{\Delta C}_{P}}{T}dT$ | Eq. S6 |
| $\Delta G_{T_{2}}^{0}=\Delta H-T_{2}S$ | Eq. S7 |

Through stoichiometric analysis, we constructed the chemical reaction equation and subsequently calculated the Gibbs free energy change (*ΔG*) for the reaction process, as detailed in Equations S8-S9.

| $\Delta G_{T_{2}}^{0}=\sum\Delta G_{f}^{0}\left( products \right)-\sum\Delta G_{f}^{0}\left( reactors \right)$ | Eq. S8 |
| --- | --- |
| $\Delta G_{T_{2}}^{0}=\Delta G^{0}+RTln\frac{\prod products}{\prod reactors}$ | Eq. S9 |

ATP requirement evaluation for biomass synthesis under 40 MPa

Assuming microbial protein content represents biomass, bacterial synthesis of one amino acid requires approximately 6 high-energy molecules (e.g., ATP)^19^. Protein amino acid composition varies considerably across species and issues, primarily determined by protein type and functions. Statistical analyses indicate that bacterial proteins typically comprise ~350 amino acids^20^. Under the known 40 MPa pressure conditions, our measurements showed a maximum protein concentration of 115 µg/ml in a 16 mL culture volume. We calculated the total protein molar quantity and corresponding ATP consumption using Eq. S10:

| $Moles of protein=\frac{Protein mass (g)}{Molecular weight (\frac{g}{mol})}$ | Eq. S10 |
| --- | --- |

This yielded an estimated ATP requirement of ~7.3 mM. Considering glycolysis produces 2 ATP molecules per glucose molecule, the fermentation-derived energy at 40 MPa was 8.2 mM, exceeding the ATP demand for biomass synthesis. Therefore, the energy surplus confirms adequate metabolic capacity to sustain biomass synthesis pathways, ensuring microbial survival under these pressure conditions.

**Discussion**

Contribution of Fe(III)-containing minerals in microbial tolerance of HHP

Iron minerals, widespread in diverse environments, play crucial roles on the fate of organic carbon. Our work offers novel insights into how ferric iron minerals support microbial growth under HHP. The conventional theory regards ferric iron minerals in fermentative iron reduction as merely incidental “electron sinks” that receive excess electrons from organic substrates, because microbial iron reduction typically contributes <5% of the organism’s total energy generation^5^. In contrast, our work suggests that the amended ferrihydrite can actively modify electron and carbon fluxes as the “electron sink”, which ultimately enhances microbial growth under HHP.

While thermodynamic modeling predicts increased favorability of microbial iron reduction under elevated hydrostatic pressures^21^, our deduction shows that the Gibbs free energy changes across our experimental pressure range are not significant. Instead, the observed thermodynamic difference at higher pressures primarily relates to altered stoichiometry between iron reduction and glucose decomposition (Table S2 and Fig. S3).

As redox-active compounds with a broad range of redox potentials, iron minerals can store electrons from electron donors when readily available electron acceptors are scarce^22^. In our system, the supplemented ferrihydrite may receive the reducing equivalents from NADH or H_2_, which simultaneously recycles NADH while increasing acetyl-CoA production. Consequently, although the glucose consumption at 40 MPa was much lower than that at lower pressures, strain Z6 catalyzes more efficient carbon flux and energy generation through enhanced ferrihydrite reduction. This mechanism explains our observation of comparable maximum protein concentrations across pressure conditions despite different metabolic activity (Figs. S2 and S3).

Membrane adaptation to high hydrostatic pressures

The cell membrane represents one of the most pressure-sensitive cellular components in microorganisms. To counteract hydrostatic pressure stress, organisms modulate their PLFA composition to decrease membrane lipid fluidity and limit molecular transport and diffusion^23-25^. Such adaptation occurs through two primary mechanisms: (1) homeoviscous adaptation, which maintains membrane viscosity through fatty acid compositional changes; (2) homeophasic adaptation, which preserves membrane permeability by sustaining the liquid-crystalline state of lipids and bilayer structure^26^.

Strain Z6 resists high pressure through decreased lipid saturation (Table S1 and Fig. 1C), which aligns with many marine microorganisms^24, 27-29^. Delong and Yayanos (1985, 1986) established that the monounsaturated-to-polyunsaturated PLFA ratio in marine microorganisms correlated positively with their isolation depth, which reflected their native hydrostatic pressure environments^24, 27^. For example, *Shewanella sp*. strain DB21MT-2 grown under high hydrostatic pressure shows higher monounsaturated PLFAs content than *Shewanella* *benthica* from lower-pressure habitats^24, 27-29^. Increasing mono- and polyunsaturated PLFAs enhances the membrane disorder, thereby increasing membrane fluidity and preventing pressure-induced lipid layer aggregation under high pressure stress^30^.

In addition, strain Z6 exhibits shortened and branched-chain PLFAs under extreme pressure (Fig. 1C). Compared to straight-chain counterparts, branched-chain PLFAs reduce melting temperatures and prevent crystalline structure formation between acyl chains, thereby increasing cell membrane fluidity^25, 31^. Longer acyl chains, conversely penetrate deeper into the phospholipid bilayer, strengthening interlayer interactions between layers, which imparts rigidity of the cell membrane^32^. While bacterial fluidity maintenance strategies vary, fatty acid saturation exerts greater influence on membrane rigidity than chain length in strain Z6^23, 33^. Collectively, these modifications highlight the critical role of PLFA remodeling in microbial high-pressure adaptation^30^.

Typical and novel pressure adaptation strategies implied by transcriptomics

Transcriptomic analysis of strain Z6 under different hydrostatic pressures suggests both constant and pressure-dependent adaptation strategies (Figs. 1E and S5). We categorize these as general and conditional strategies, respectively, to reflect their distinct operational contexts. To validate the robustness of the transcriptomic data, representative genes (*afu*, U472_12305; *grx*, U472_03755) were selected and quantified via reverse transcription-quantitative PCR (qRT-PCR) (R^2^=0.88 and 0.97, respectively) under various hydrostatic pressure treatments (10–40 MPa). The qRT-PCR results showed strong correlations with the RNA-Seq profiles (Fig. S7).

*General pressure adaptation strategies*

Strain Z6 employs general pressure adaptation strategies involving antioxidant defense systems, DNA/protein repair, ion transport regulation, compatible solute accumulation, membrane lipids, and EPS metabolism (Fig. S5 and Table S4). The antioxidant defense system represents a universal bacterial stress response, including cold, acid, heat, heavy metals, salinity, and UV radiation^34^. For example, the deep-sea bacterium *S. piezotolerans* WP3 upregulates genes encoding glutathione peroxidase (*gpx*), transcriptional regulator (e.g., *oxyR*), and catalase (e.g., *katE*) under high-pressure and low-temperature conditions^35^. Similarly, *Sporosarcina psychrophila* enhances catalase and thiol oxidase activity with MgCl_2_ supplement to alleviate oxidative stress under otherwise prohibitive pressure conditions^10^. For strain Z6, its HHP adaptation appears partially mediated through SOS response and DNA repair systems, as evidenced by overexpression of the associated genes (Fig. S5). This comprehensive defense strategy enables survival across varying pressure regimes while maintaining cellular integrity.

Ion transport and compatible solutes accumulation represent additional crucial mechanisms for pressure adaptation. Iron plays a dual role in microbial physiology. While it serves as a cofactor for oxidative phosphorylation and DNA synthesis^36, 37^, excessive intracellular iron can generate harmful free radicals. The balance of iron is primarily regulated by ferric uptake regulator (Fur) protein, which suppresses transcription of most metal-regulated genes under iron-rich conditions^36^. For example, *Sporosarcina psychrophila* upregulates iron transport systems and Fur expression to mitigate the oxidative stress from Fenton reactions under stress conditions^10^.

Piezophiles synthesize and accumulate organic compatible solutes as a universal response to extreme environmental challenges (e.g., temperature fluctuation, salinity changes, and pH variation)^38, 39^. Under osmotic stress, microorganisms commonly accumulate glycine and betaine in response to elevated osmotic conditions. For example, glycine and betaine accumulate in *S. psychrophila* DSM 6497 to overcome the osmotic pressure following MgCl_2_ supplementation^10^. Many piezophiles, including *Desulfovibrio hydrothermalis* AM13 and *D. piezophilus* C1TLV30T, demonstrate pressure-dependent increases in intracellular glutamate concentrations, confirming its role as a pressure-responsive compatible solute^38, 40, 41^. For *Nautilia sp.* strain PV-1, isolated from a deep-sea hydrothermal vent, expression of the glutamine ABC transporter was also upregulated under high-temperature and high-pressure conditions^42^. In addition to osmotic regulation, the compatible solutes may also function as antioxidants to scavenge free radicals and reactive oxygen species while stabilizing biomolecular structures to protect cellular metabolism under pressure stress^43, 44^.

In response to membrane lipid modification (Fig. 1C), upregulation of many genes related to membrane lipid synthesis is observed under HHP. Specifically, the acyltransferase-encoding genes *lpxJ* and *lpxL* are upregulated, which are associated with lipopolysaccharide biosynthesis and bacterial outer membrane stability, respectively^45^. Additionally, the genes related to EPS biosynthesis and biofilm formation show increased expression. For example, the upregulated *tamB* gene encodes the TamB membrane protein, a component of the transfer and assembly module that facilitates adhesin protein secretion and promotes biofilm formation^46^. For Gram-negative bacteria, the TonB system comprising TonB, ExbB, and ExbD proteins anchors to the cytoplasmic membrane and utilizes proton motive force (PMF) to power transport of various molecules (e.g., ferric iron carriers, vitamin B_12_, heme, and siderophores) across the outer membrane^47, 48^. This transport process requires ATP-derived energy. Interestingly, strain Z6 downregulates these TonB system genes, which may conserve limited cellular energy generated for survival under high-pressure conditions.

*Conditional pressure adaptation strategies*

Strain Z6 demonstrates some condition-specific adaptation strategies that are upregulated only under certain pressure conditions. At 10–30 MPa, the expression of genes encoding chemotaxis proteins and histidine kinase involved in chemotaxis peaks (Table S5). For many marine microorganisms, motility serves a critical function for nutrient acquisition and predator avoidance^49, 50^. The flagellar system appears particularly important for piezophilic growth. For instance, expression of the genes for motility of the obligate piezophile *Pyrococcus yayanosii* is upregulated when the pressures exceed its optimal range^51^. For the piezophilic *Desulfovibrio alaskensis*, a mutation in flagellar biosynthesis genes impaired its motility and growth at high pressures^52^. The flagellar system of strain Z6 comprises the proteins for polar flagella and controls cellular motility. In addition, nearly all its genes associated with flagellar synthesis and regulation were downregulated at elevated pressure (Table S6). Meanwhile, natural competence is the ability for a cell to take up exogenous DNA from the ambient environment for transformation^53^. Strain Z6 overexpresses the competence genes at 20–30 MPa, potentially enabling nutrient recycling from lysed cells through natural transformation. This energy-saving strategy mainly helps maintain essential metabolisms under pressure stress^54^.

Notably, strain Z6 exhibits maximal upregulation of phosphotransferase system (PTS) and cold-shock response genes at 40 MPa (Fig. 1E). The PTS mediates transport of sugars and their derivatives via phosphorylation cascades^55^. In addition to regulating central carbon and nitrogen metabolism, it also mediates iron/potassium homeostasis and stress responses. Studies on *E. coli* suggest that PTS may modulate oxidative stress tolerance through cAMP regulation^56, 57^. Alternatively, it may exert genetic regulation on the non- or slow-growing cells (persisters) exposed to lethal concentrations of bacteriocin^58^. PTS may also enhance environmental sensing to help bacteria evade lethal external stimuli. For instance, microbial sensitivity to chemical stimuli on long-term spaceflight may be associated with a significant upregulation of the PTS gene expression^59^. These PTS adaptations likely aid survival under extreme pressure. Furthermore, the cold-shock protein response parallels findings in deep-sea bacterium *S. piezotolerans* WP3, which shares a similar adaptation strategy to both high pressure and low temperature^35^. This consistency supports the known similarities between high-pressure and cold stress responses^35^.

Table S1. The relative content of phospholipid fatty acids under different pressures.

| **PLFA compound** | **Relative content (%)** | | | | |
| --- | --- | --- | --- | --- | --- |
|  | **0.1 MPa** | **10 MPa** | **20 MPa** | **30 MPa** | **40 MPa** |
| C_15_H_28_O_2_ | 2 | 3 | 3 | 3 | 4 |
| C_15_H_30_O_2_ | 19 | 17 | 14 | 12 | 16 |
| C_17_H_32_O_2_ | 33 | 34 | 35 | 38 | 32 |
| C_17_H_34_O_2_ | 39 | 36 | 38 | 36 | 17 |
| C_19_H_36_O_3_ | 1 | 2 | 2 | 2 | 2 |
| C_19_H_36_O_2_ | 1 | 1 | 1 | 1 | 7 |
| C_19_H_38_O_2_ | 1 | 1 | 1 | 1 | 9 |
| C_17_H_32_O_2_ | 1 | 1 | 1 | 2 | 3 |

Table S2. Mass balance and thermodynamic analysis of fermentative iron reduction by strain Z6 under different pressure conditions.

| **Rxn.** | **Pressure (MPa)** | **Stoichiometry of the Reactions and Gibbs Free Energy*^a^*^,^ *^b^*** | | | $\boldsymbol{\Delta G}_{\boldsymbol{310.15}\boldsymbol{K}}\boldsymbol{(kJ)}$***^c^*** | | $\boldsymbol{\Delta G}_{\boldsymbol{310.15}\boldsymbol{K, int.}}\boldsymbol{(kJ)}$*^d^* | | | | |  |
| --- | --- | --- | --- | --- | --- | --- | --- | --- | --- | --- | --- | --- |
| 1 | 0.1 | C_6_H_12_O_6_+4/5Fe(OH)_3_+19/90H^+^→8/9HCOO^-^+1/2CH_3_COO-+C_2_H_5_OH+6/5CO_2_+41/45C_Bio_+8/9H_2_ | | | -303.15 | | -459 | | | | |  |
|  |  | +4/5Fe^2+^+20/9H_2_O | | |  | |  | | | | |  |
| 1-1 |  | I: C_6_H_12_O_6_+7/15H_2_O→8/9HCOO^-^+1/2CH_3_COO^-^+C_2_H_5_OH+6/5CO_2_+41/45C_Bio_+25/18H^+^+58/45H_2_ | | | -200.36 | |  | | | | |  |
| 1-2 |  | II: 4/5Fe(OH)_3_+8/5H^+^+2/5H_2_→4/5Fe^2+^+12/5H_2_O | | | -104.46 | |  | | | | |  |
|  |  |  | | |  | |  | | | | |  |
| 2 | 10-30 | C_6_H_12_O_6_+4/5Fe(OH)_3_+8/15H^+^→2/3HCOO^-^+2/5CH_3_COO^-^+C_2_H_5_OH+6/5CO_2_+4/3C_Bio_+2/3H_2_ | | | -343.51 | | -507.76 | | | | |  |
|  |  | +4/5Fe^2+^+43/15H_2_O | | |  | |  | | | | |  |
| 2-1 |  | I: C_6_H_12_O_6_→2/3HCOO^-^+2/5CH_3_COO^-^+C_2_H_5_OH+6/5CO_2_+4/3C_Bio_+16/15H_2_+7/15H_2_O | | | -242.39 | |  | | | | |  |
| 2-2 |  | II: 4/5Fe(OH)_3_+8/5H^+^+2/5H_2_→4/5Fe^2+^+12/5H_2_O | | | -104.46 | |  | | | | |  |
|  |  |  |  |  | | | | | | | | |
| 3 | 40 | C_6_H_12_O_6_+3/2Fe(OH)_3_+29/15H^+^→2/3HCOO^-^+2/5CH_3_COO^-^+1/2C_2_H_5_OH+12/5CO_2_+17/15C_Bio_ | | | | -361.12 | | -550.12 | | |  |  |
|  |  | +253/60H_2_+3/2Fe^2+^+46/15H_2_O | | | |  | |  |  |  |  |  |
| 3-1 |  | I: C_6_H_12_O_6_+43/30H_2_O→2/3HCOO^-^+2/5CH_3_COO^-^+1/2C_2_H_5_OH+12/5CO_2_+17/15C_Bio_+149/30H_2_ | | | | -158.10 | |  | | |  |  |
| 3-2 |  | II: 3/2Fe(OH)_3_+3H^+^+3/4H_2_→3/2Fe^2+^+9/2H_2_O | | | | -199.23 |  | | |  |  |  |

*^a^*The chemical equations were developed based on the changes in substrate and product concentrations, and the biomass was determined as the maximum protein concentrations using the BCA assay; *^b^*Each chemical equation was divided into two sub-equations I and II, based on our previous study showing that strain Z6 only used H_2_ generated from glycolysis of glucose as the electron donor for dissimilatory iron reduction^5^. *^c^*The Gibbs Free Energy at 37°C was modified from that under standard conditions based on the method as detailed in the SI. *^d^* Calculation of the Gibbs Free Energy at the initial of the experiments was based on the assumption that the concentration of the glucose was 0.02 mM.

Table S3. Summary of transcriptomic sequencing results*^a^*.

| **Sample** | **Total clean reads (Gb)** | **Clean reads ratio (%)** | **GC content (%)** | **Number of expressed genes** |
| --- | --- | --- | --- | --- |
| 0.1 MPa | 2.00 | 98.72 | 42.16 | 2700 |
| 10 MPa | 2.43 | 99.60 | 43.84 | 3040 |
| 20 MPa | 2.16 | 98.16 | 47.99 | 3026 |
| 30 MPa | 2.29 | 98.2 | 37.46 | 3026 |
| 40 MPa | 2.15 | 98.17 | 38.97 | 3027 |

*^a.^* All the samples were analyzed in triplicates and the values were calculated as the average of the replicates. The standard deviation for the replicates was < 8.2 % for total clean reads and <3.3 % for other parameters.

Table S8. Primers for RT-PCR were used in this study.

| **Primer** | **Targeted gene** | **Primer sequence** | **T_m_ (°C)** |
| --- | --- | --- | --- |
| afuB_1F | *afuB* | CCTGATAGTTTTGTTCTAGAAAGGG | 57 |
| afuB_1R |  | GCAGGGGGTAGTTTGGTTG | 58 |
| grx_2F | *grx* | AGTTGTCAGCAACAGTTAG | 53 |
| grx_2R |  | TGGTTTTTTAGAGGGGGTATAAAATAC | 57.5 |
|  |  |  |  |

Figure S1. Concentrations of 0.5 N HCl-extractable Fe(II) in the abiotic controls under different pressure conditions. The biological experimental group at 0.1 MPa was illustrated in red as the reference.


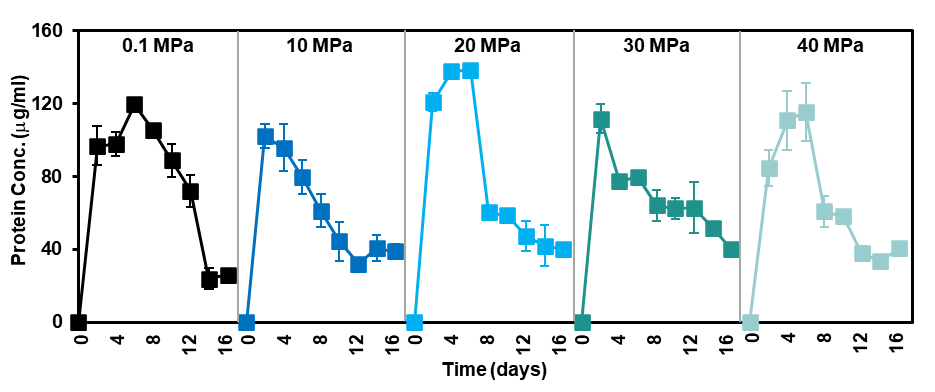
Figure S2. The protein concentrations in the iron-reducing cultures incubated under the pressure conditions ranging from 0.1 to 40 MPa. 10 mM of glucose and 15 mM ferrihydrite were amended as the carbon source and electron acceptor, respectively. Under individual pressure condition, a total of 16 replicates were prepared and 2 of them were sacrificed for analyses at each time point. The error bars indicated standard deviation of replicates.


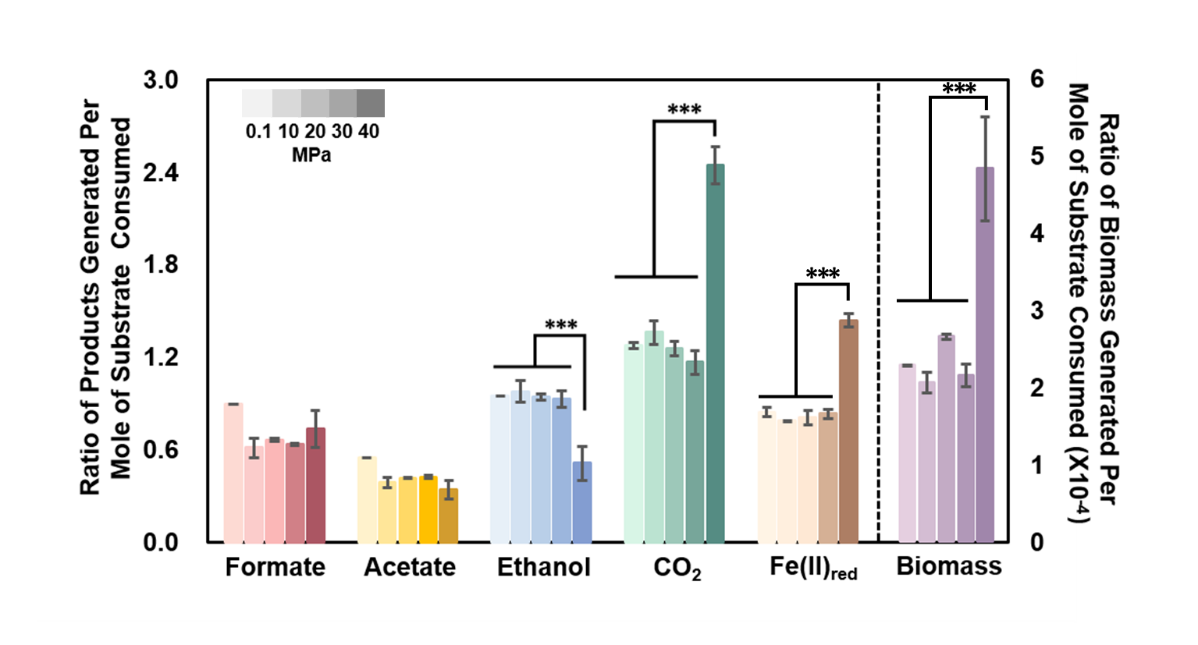
Figure S3. The ratios of products generated per mole of glucose consumed by strain Z6 on Day 16 under different hydrostatic pressures (i.e., 0.1, 10, 20, 30, or 40 MPa). All the samples were prepared in triplicates, and the error bars indicate standard deviation of the replicates. ***: *p*-value <0.001 for *t*-test.


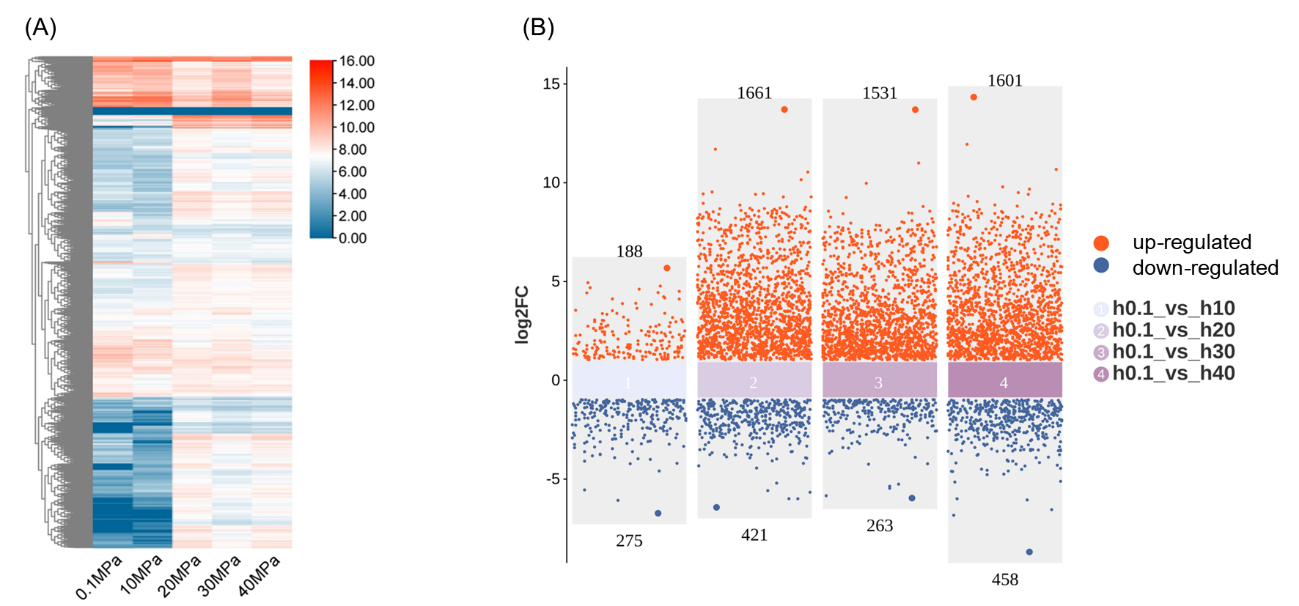
Figure S4. The log_10_(TPM+1) values for gene expression under different pressure condition (A) and the differentially expressed genes (DEGs) under various pressure conditions based on the cutoff log_2_|fold change[FC]| ≥1 (B). TPM means transcripts per million. The fold change (FC) value for each gene was calculated by dividing its TPM under the gene under specific pressure conditions by that under 0.1 MPa.


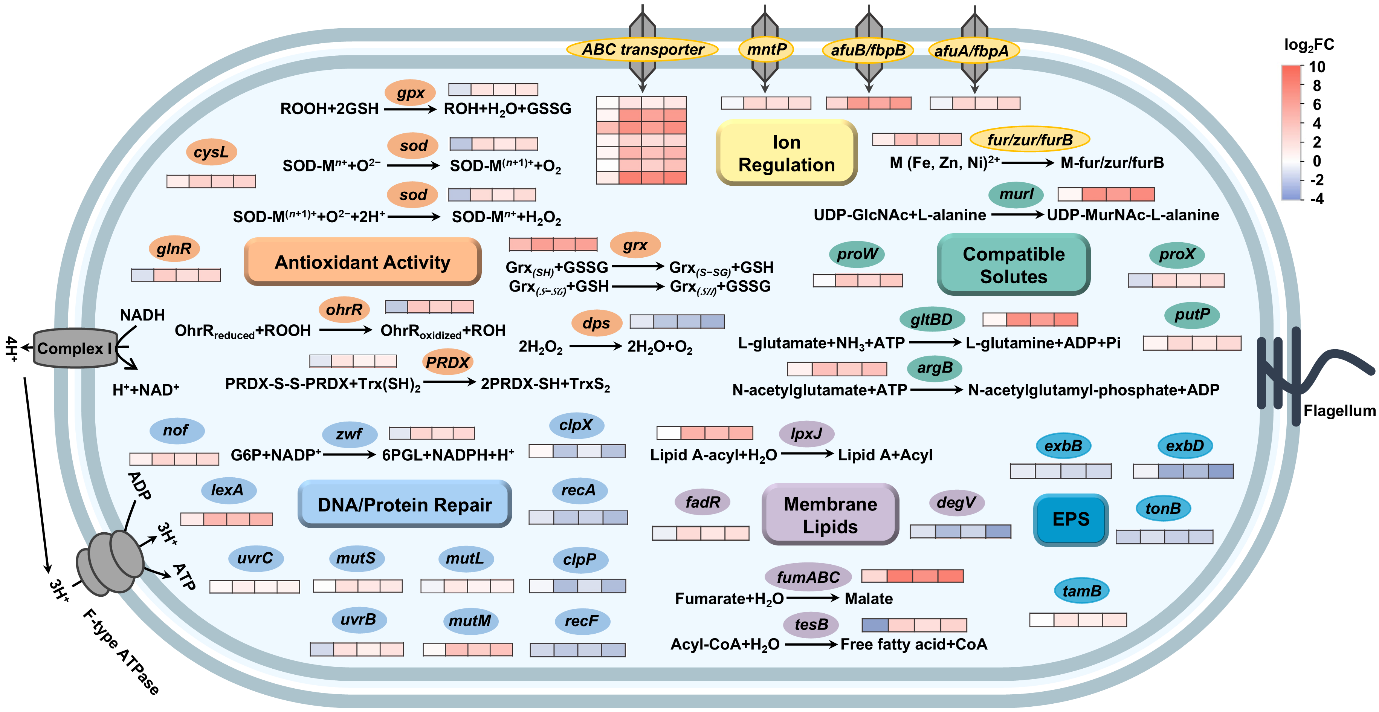
Figure S5. General adaptation strategies by strain Z6 under gradient hydrostatic pressures. The heatmaps display the fold change (FC) in differentially expressed genes (DEGs) across pressure conditions (10, 20, 30 and 40 MPa, from left to right), calculated as the ratios of their TPM under pressure versus the 0.1 MPa control. Only the genes with |log_2_FC| ≥ 1 are included. The information about the gene annotation is detailed in supplementary Table S4 . The names of the genes and their descriptions are as follows: *ABC-2.A*: ABC transporter ATP-binding protein; *ABC.cd.a*: ABC transporter; *ABC.cd.p*: ABC transporter permease; *ABC.fev.p*: iron ABC transporter; *afuB/fbpA*: Fe(3+) ABC transporter substrate-binding protein; *afuB/fbpB*: iron ABC transporter permease; *argB*: acetylglutamate kinase; *cheY*: two-component system: chemotaxis family: response regulator CheY; *clpP*: ATP-dependent Clp protease: protease subunit; *clpX*: ATP-dependent Clp protease ATP-binding subunit ClpX; *comAEF*: competence protein; *cspA*: cold-shock protein; *celB/chbC*: PTS lactose/cellobiose family IIC subunit; *cysL*: LysR family transcriptional regulator; *degV*: fatty acid-binding protein DegV; *dhaM*: PTS mannose transporter subunit IID; *dps*: ferritin Dps family protein; *exbB*: biopolymer transport protein ExbB; *exbD*: biopolymer transport protein ExbD; *fumABC*: fumarate hydratase; *fadR*: fatty acid biosynthesis transcriptional regulator; *ftr1*: iron permease FTR1; *fruAb*: fructose PTS system EIIB component [EC:2.7.1.202]; *fur/zur/furB*: Fur family transcriptional regulator: ferric uptake regulator; *fusA/typA*: GTPase; *glnR*: glutamine synthetase repressor; *gltB/gltD*: glutamate synthase; *grx*: glutaredoxin; *gpx*: glutathione peroxidase; *hica*: toxin Hica; *hicb*: type II toxin-antitoxin system HicB family antitoxin; *hk*: histidine kinase; *k07058*: membrane protein; *lexA*: repressor LexA; *lpxJ*: Kdo2-lipid IVA 3’ secondary acyltransferase; *manX/mnsA*: PTS mannitol transporter subunit IIA; *mcp*: chemotaxis protein; *mntP*: manganese efflux pump family protein; *mutL*: DNA mismatch repair protein MutL; *mutM/fpg*: formamidopyrimidine-DNA glycosylase; *mutS*: DNA mismatch repair protein MutS; *murl*: glutamate racemase; *nfo*: deoxyribonuclease IV; *ohrR*: organic hydroperoxide resistance regulator; *PRDX*: peroxiredoxin; *proX*: glycine betaine/proline transport system substrate-binding protein; *proW*: glycine betaine/proline transport system permease protein; *p19/ftrA*: iron transporter; *putP*: sodium/proline symporter; *recA*: recombination protein RecA; *recF*: DNA replication and repair protein RecF; *RpoS/OxyR*: stress responsive protein; *sod*: superoxide dismutase; *tamB*: translocation and assembly module TamB; *TesB*: acyl-CoA thioesterase; *tetR/acrR*: TetR/AcrR family transcriptional regulator; *tonB*: periplasmic protein TonB; *ulaB/sgaB*: PTS ascorbate transporter subunit IIB; *uvrB*: excinuclease ABC subunit B; *uvrC*: excinuclease ABC subunit C; *zwf*: glucose-6-phosphate 1-dehydrogenase.


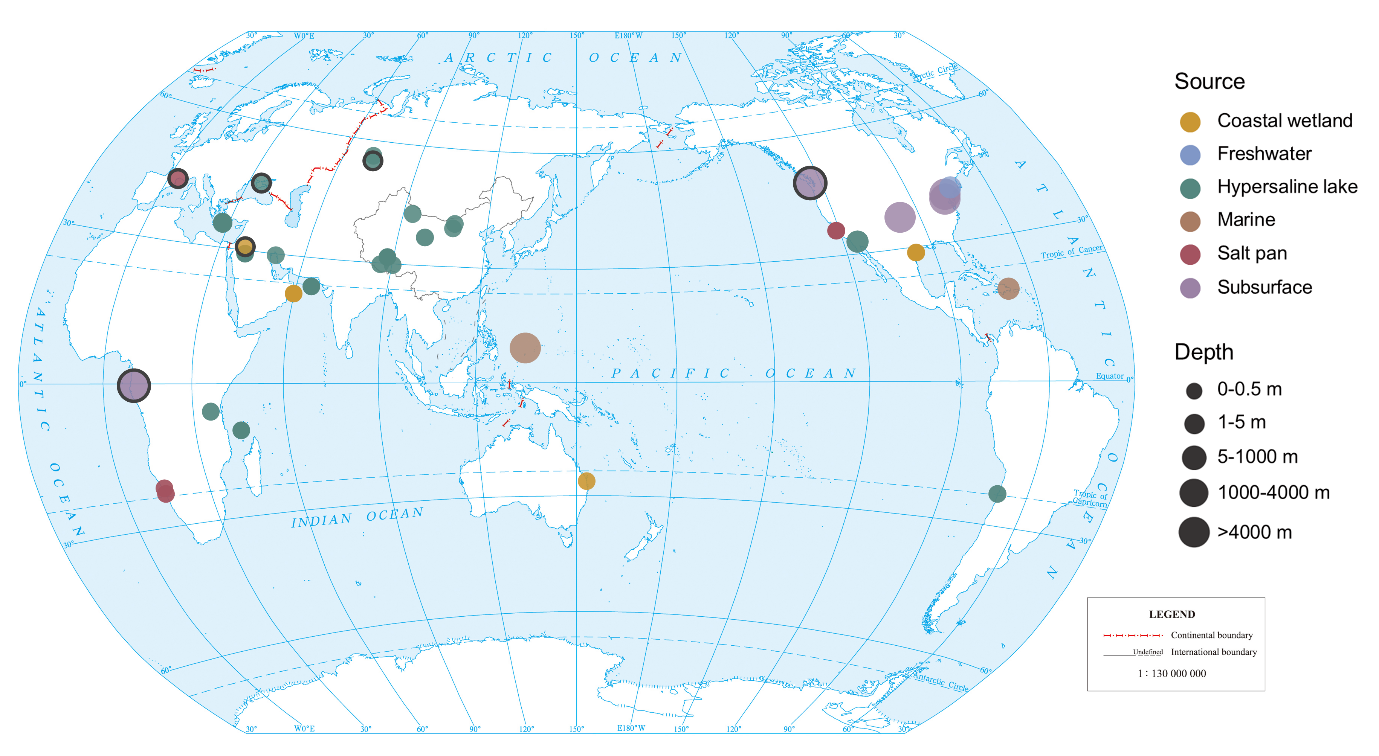
Figure S6. Global distribution of Genus *Orenia*. The points on the map indicates the geographical coordinate of the sites, from which either an *Orenia* species was isolated (n=6) or detected in the metagenomes of environmental samples (n=83). The circles with edge represent the sampling sites with *Orenia* pure cultures, while those without edge denote detection of *Orenia* species in metagenomic samples. The metagenome-derived *Orenia* species were detected based on Sandpiper^60^. For some samples from the same or geographically closed sites, their labels may overlap. The information of the metagenoms from which *Orenia* species were detected is listed in Table S7. Approval number for the map: GS(2016)2957.


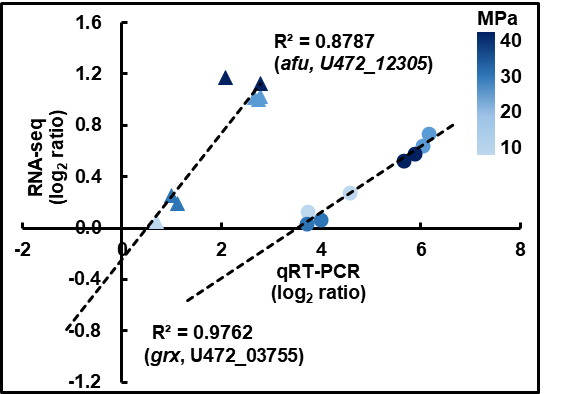
Figure S7. The RNA-Seq expression data were confirmed for selection of representative genes (*afu*, U472_12305; *grx*, U472_03755) using reverse transcription quantitative PCR (RT-qPCR) across the various pressure treatments (10–40 MPa). The x-axis indicates the log2-fold change compared with those at 0.1 MPa according to the RT-PCR results, the y-axis represents the log2-fold change compared with those at 0.1 MPa according to the RNA-Seq results. R^2^ indicates the R-square of linear regression.

**References**

(1) Schwertmann U, Stanjek H, Becher H-H. Long-term in vitro transformation of 2-line ferrihydrite to goethite/hematite at 4, 10, 15 and 25°C. Clay Miner. 2004;39 (4):433-438.

(2) Schwertmann U, Wagner FE, Knicker HE. Ferrihydrite-humic associations: magnetic hyperfine interactions. Soil Sci. Soc. Am. J. 2005;69:1009-1015.

(3) Dong Y, Sanford RA, Boyanov MI, Kemner KM, Flynn TM, O'Loughlin EJ, et al. *Orenia metallireducens* sp. nov. strain Z6, a novel metal-reducing member of the phylum firmicutes from the deep subsurface. Appl. Environ. Microbiol. 2016;82 (21):6440-6453.

(4) Dong Y, Kumar CG, Chia N, Kim PJ, Miller PA, Price ND, et al. *Halomonas sulfidaeris*-dominated microbial community inhabits a 1.8 km-deep subsurface Cambrian Sandstone reservoir. Environ. Microbiol. 2014;16 (6):1695-1708.

(5) Dong Y, Sanford RA, Chang YJ, McInerney MJ, Fouke BW. Hematite reduction buffers acid generation and enhances nutrient uptake by a fermentative iron reducing bacterium, *Orenia metallireducens* strain Z6. Environ. Sci. Technol. 2017;51 (1):232-242.

(6) Gibbs CR. Characterization and application of FerroZine iron reagent as a ferrous iron indicator. Anal. Chem. 1976;48 (8):1197-1201.

(7) Blake JD, Clarke ML, Richards GN. Determination of organic acids in sugar cane process juice by high-performance liquid chromatography: improved resolution using dual Aminex HPX-87H cation-exchange columns equilibrated to different temperatures. J. Chromatogr. 1987;398:265-277.

(8) White DC, Davis WM, Nickels JS, King JD, Bobbie RJ. Determination of the sedimentary microbial biomass by extractible lipid phosphate. Oecologia*.* 1979;40 (1):51-62.

(9) Frostegård Å, Tunlid A, Bååth E. Microbial biomass measured as total lipid phosphate in soils of different organic content. J. Microbiol. Methods*.* 1991;14 (3):151-163.

(10) Wang H, Zhang Y, Bartlett DH, Xiao X. Transcriptomic analysis reveals common adaptation mechanisms under different stresses for moderately piezophilic bacteria. Microb. Ecol. 2021;81 (3):617-629.

(11) Viollier E, Inglett PW, Hunter K, Roychoudhury AN, Van Cappellen P. The ferrozine method revisited: Fe(II)/Fe(III) determination in natural waters. Appl. Geochem. 2000;15 (6):785-790.

(12) Dowling NJE, Widdel F, White DC. Phospholipid ester-linked fatty ccid biomarkers of acetate-oxidizing sulphate-reducers and other sulphide-forming bacteria. Microbiology*.* 1986;132 (7):1815-1825.

(13) Jensen NJ, Gross ML. Mass spectrometry methods for structural determination and analysis of fatty acids. Mass Spectrom. Rev. 1987;6 (4):497-536.

(14) Bolger AM, Lohse M, Usadel B. Trimmomatic: a flexible trimmer for Illumina sequence data. Bioinformatics*.* 2014;30 (15):2114-2120.

(15) Tjaden B. A computational system for identifying operons based on RNA-seq data. Methods*.* 2020;176:62-70.

(16) McCarthy DJ, Chen Y, Smyth GK. Differential expression analysis of multifactor RNA-Seq experiments with respect to biological variation. Nucleic Acids Res. 2012;40 (10):4288-4297.

(17) Klopfenstein DV, Zhang L, Pedersen BS, Ramírez F, Warwick Vesztrocy A, Naldi A, et al. GOATOOLS: A Python library for Gene Ontology analyses. Sci. Rep. 2018;8 (1):10872.

(18) Bu D, Luo H, Huo P, Wang Z, Zhang S, He Z, et al. KOBAS-i: intelligent prioritization and exploratory visualization of biological functions for gene enrichment analysis. Nucleic Acids Res. 2021;49 (W1):W317-W325.

(19) Akashi H, Gojobori T. Metabolic efficiency and amino acid composition in the proteomes of *Escherichia coli* and *Bacillus subtilis* Proc. Natl. Acad. Sci. U. S. A. 2002;99 (6):3695-3700.

(20) Santos-Magalhaes NS, Oliveira HMd. Of protein size and genomes. WSEAS Transactions on Mathematics and Computers in Biology and Biomedicine*.* 2006;3(2) (133–138).

(21) Fang J, Zhang L, Bazylinski DA. Deep-sea piezosphere and piezophiles: geomicrobiology and biogeochemistry. Trends Microbiol. 2010;18 (9):413-422.

(22) Cui S, Wang R, Chen Q, Pugliese L, Wu S. Geobatteries in environmental biogeochemistry: Electron transfer and utilization. Environ. Sci. Ecotechnology*.* 2024;22:100446.

(23) Scoma A, Garrido-Amador P, Nielsen SD, Røy H, Kjeldsen KU. The polyextremophilic bacterium *Clostridium paradoxum* attains piezophilic traits by modulating its energy metabolism and cell membrane composition. Appl. Environ. Microbiol. 2019;85 (15):e00802-00819.

(24) Delong EF, Yayanos AA. Biochemical function and ecological significance of novel bacterial lipids in deep-sea procaryotes. Appl. Environ. Microbiol. 1986;51 (4):730-737.

(25) Diomandé SE, Nguyen-The C, Guinebretière MH, Broussolle V, Brillard J. Role of fatty acids in Bacillus environmental adaptation. Front. Microbiol. 2015;6:813.

(26) McElhaney RN. The relationship between membrane lipid fluidity and phase state and the ability of bacteria and mycoplasmas to grow and survive at various temperatures. In *Membrane Fluidity*, Kates, M., Manson, L. A. Eds.; Springer US, 1984; pp 249-278.

(27) DeLong EF, Yayanos AA. Adaptation of the membrane lipids of a deep-sea bacterium to changes in hydrostatic pressure. Science*.* 1985;228 (4703):1101-1103.

(28) Allen EE, Facciotti D, Bartlett DH. Monounsaturated but not polyunsaturated fatty acids are required for growth of the deep-sea bacterium *Photobacterium profundum* SS9 at high pressure and low temperature. Appl. Environ. Microbiol. 1999;65 (4):1710-1720.

(29) Nogi Y, Kato C, Horikoshi K. Taxonomic studies of deep-sea barophilic Shewanella strains and description of *Shewanella violacea* sp. nov. Arch. Microbiol. 1998;170 (5):331-338.

(30) Oger PM, Jebbar M. The many ways of coping with pressure. Res. Microbiol. 2010;161 (10):799-809.

(31) Denich TJ, Beaudette LA, Lee H, Trevors JT. Fluorescent methods to study DNA, RNA, proteins and cytoplasmic membrane polarization in the pentachlorophenol-mineralizing bacterium *Sphingomonas* sp. UG30 during nutrient starvation in water. J. Fluoresc. 2005;15 (2):143-151.

(32) Rowan-Nash AD, Korry BJ, Mylonakis E, Belenky P. Cross-domain and viral interactions in the microbiome. Microbiol. Mol. Biol. Rev. 2019;83 (1).

(33) Kaneda T. Iso- and anteiso-fatty acids in bacteria: biosynthesis, function, and taxonomic significance. Microbiol. Mol. Biol. Rev. 1991;55 (2):288-302.

(34) Scheffer G, Gieg LM. The mystery of piezophiles: understudied microorganisms from the deep, dark subsurface. Microorganisms*.* 2023;11 (7):1629.

(35) Xie Z, Jian H, Jin Z, Xiao X. Enhancing the adaptability of the deep-sea bacterium *Shewanella piezotolerans* WP3 to high pressure and low temperature by experimental evolution under H_2_O_2_ stress. Appl. Environ. Microbiol. 2017;84 (5):e02342-02317.

(36) Escolar L, Perez-Martin JD, Lorenzo Vd. Opening the iron box: transcriptional metalloregulation by the fur protein. J. Bacteriol. 1999;181 (20):6223-6229.

(37) Hantke K. Iron and metal regulation in bacteria. Curr. Opin. Microbiol. 2001;4 (2):172-177.

(38) Ambily Nath IV, Loka Bharathi PA. Diversity in transcripts and translational pattern of stress proteins in marine extremophiles. Extremophiles*.* 2011;15 (2):129-153.

(39) Ambily Nath I, Loka Bharathi P. Diversity in transcripts and translational pattern of stress proteins in marine extremophiles. Extremophiles*.* 2011;15:129-153.

(40) Amrani A, van Helden J, Bergon A, Aouane A, Ben Hania W, Tamburini C, et al. Deciphering the adaptation strategies of *Desulfovibrio piezophilus* to hydrostatic pressure through metabolic and transcriptional analyses. Environ. Microbiol. Rep. 2016;8 (4):520-526.

(41) Amrani A, Bergon A, Holota H, Tamburini C, Garel M, Ollivier B, et al. Transcriptomics reveal several gene expression patterns in the piezophile *Desulfovibrio hydrothermalis* in response to hydrostatic pressure. PLoS One*.* 2014;9 (9):e106831.

(42) Smedile F, Foustoukos DI, Patwardhan S, Mullane K, Schlegel I, Adams MW, et al. Adaptations to high pressure of Nautilia sp. strain PV-1, a piezophilic Campylobacterium (aka Epsilonproteobacterium) isolated from a deep-sea hydrothermal vent. Environ. Microbiol. 2022;24 (12):6164-6183.

(43) Yancey PH. Organic osmolytes as compatible, metabolic and counteracting cytoprotectants in high osmolarity and other stresses. J. Exp. Biol. 2005;208 (Pt 15):2819-2830.

(44) Yancey, P. H. Organic osmolytes as compatible, metabolic and counteracting cytoprotectants in high osmolarity and other stresses. Journal of Experimental Biology*.* 2005;208 (15):2819-2830.

(45) Rubin EJ, O'Brien JP, Ivanov PL, Brodbelt JS, Trent MS. Identification of a broad family of lipid A late acyltransferases with non-canonical substrate specificity. Mol. Microbiol. 2014;91 (5):887-899.

(46) Lohmiller S, Hantke K, Patzer SI, Braun V. TonB-dependent maltose transport by Caulobacter crescentus. Microbiology*.* 2008;154 (Pt 6):1748-1754.

(47) Schauer K, Rodionov DA, de Reuse H. New substrates for TonB-dependent transport: do we only see the 'tip of the iceberg'? Trends Biochem. Sci. 2008;33 (7):330-338.

(48) Braun V. Energy-coupled transport and signal transduction through the gram-negative outer membrane via TonB-ExbB-ExbD-dependent receptor proteins. FEMS Microbiol. Rev. 1995;16 (4):295-307.

(49) Hans-Peter G, Lasse R, Farooq A. Bacterial motility in the sea and its ecological implications. Aquat. Microb. Ecol. 2001;25 (3):247-258.

(50) Carsten M, Jens B, Hartmut A, Klaus JÂr. Role of bacterial phenotypic traits in selective feeding of the heterotrophic nanoflagellate *Spumella* sp. Aquat. Microb. Ecol. 2002;27 (2):137-148.

(51) Michoud G, Jebbar M. High hydrostatic pressure adaptive strategies in an obligate piezophile *Pyrococcus yayanosii*. Sci. Rep. 2016;6 (1):27289.

(52) Williamson AJ, Carlson HK, Kuehl JV, Huang LL, Iavarone AT, Deutschbauer A, et al. Dissimilatory sulfate reduction under high pressure by *Desulfovibrio alaskensis* G20. Front. Microbiol. 2018;9:1465.

(53) Huang M, Liu M, Huang L, Wang M, Jia R, Zhu D, et al. The activation and limitation of the bacterial natural transformation system: The function in genome evolution and stability. Microbiol. Res. 2021;252:126856.

(54) Solomon JM, Grossman AD. Who's competent and when: regulation of natural genetic competence in bacteria. Trends Genet. 1996;12 (4):150-155.

(55) Deutscher J, Aké FMD, Derkaoui M, Zébré AC, Cao TN, Bouraoui H, et al. The bacterial phosphoenolpyruvate: carbohydrate phosphotransferase system: regulation by protein phosphorylation and phosphorylation-dependent protein-protein interactions. Microbiol. Mol. Biol. Rev. 2014;78 (2):231-256.

(56) Bahr T, Lüttmann D, März W, Rak B, Görke B. Insight into bacterial phosphotransferase system-mediated signaling by interspecies transplantation of a transcriptional regulator. J. Bacteriol. 2011;193 (8):2013-2026.

(57) Lee J, Park Y-H, Kim Y-R, Seok Y-J, Lee C-R. Dephosphorylated NPr is involved in an envelope stress response of *Escherichia coli*. Microbiology*.* 2015;161 (5):1113-1123.

(58) Zeng J, Hong Y, Zhao N, Liu Q, Zhu W, Xiao L, et al. A broadly applicable, stress-mediated bacterial death pathway regulated by the phosphotransferase system (PTS) and the cAMP-Crp cascade. Proc. Natl. Acad. Sci. U. S. A. 2022;119 (23):e2118566119.

(59) Bai P, Zhang B, Zhao X, Li D, Yu Y, Zhang X, et al. Decreased metabolism and increased tolerance to extreme environments in *Staphylococcus warneri* during long-term spaceflight. Microbiologyopen*.* 2019;8 (12):e917.

(60) Woodcroft BJ, Aroney STN, Zhao R, Cunningham M, Mitchell JAM, Nurdiansyah R, et al. Comprehensive taxonomic identification of microbial species in metagenomic data using SingleM and Sandpiper. Nature Biotechnology*.* 2025.
